# Loss of *CHT3* in *Candida albicans* wild-type strains increases surface-exposed chitin and affects host-pathogen interaction

**DOI:** 10.3389/fcimb.2025.1654710

**Published:** 2025-09-05

**Authors:** María Teresa Blázquez-Muñoz, Augusto Costa-Barbosa, María Alvarado, Alexandre Mendonça, Sami Benkhellat, Manuel Vilanova, Sebastián Fernández-Sánchez, Alexandra Correia, Piet W. J. de Groot, Paula Sampaio

**Affiliations:** ^1^ Institute for Biomedicine, ETSIAMB, University of Castilla-La Mancha, Albacete, Spain; ^2^ Aquatic Research Network Associate Laboratory, Centre of Molecular and Environmental Biology, University of Minho, Braga, Portugal; ^3^ Institute of Science and Innovation for Sustainability, University of Minho, Braga, Portugal; ^4^ i3S, Institute of Health Research and Innovation, University of Porto, Porto, Portugal; ^5^ Institute of Molecular and Cell Biology, University of Porto, Porto, Portugal; ^6^ Institute of Biomedical Sciences Abel Salazar, University of Porto, Porto, Portugal

**Keywords:** *Candida albicans*, candidiasis, chitinase, host-pathogen interactions, cell separation, cell wall

## Abstract

The chitinase Cht3 plays a major role in the chitinolytic activity of the pathogenic yeast *Candida albicans* and has also been proposed as a major antigen with potential for vaccine development against systemic candidiasis. The current study aims to enhance our knowledge on the role of Cht3 in cell surface organization and virulence of *C. albicans.* To this end, *CHT3* deletion mutants generated in two wild-type genetic backgrounds (reference strain SC5314 and clinical isolate 124A) were phenotypically characterized. Absence of *CHT3* did not affect growth rate but affected cell separation of dividing yeast cells at 37 °C. Further, *cht3*Δ mutants showed enhanced levels of surface-exposed chitin and slightly increased resistance to the cell wall perturbants Calcofluor white and Congo red and the β-1,3-glucan hydrolyzing enzyme Zymolyase, while the total level of chitin appeared unaltered. Deletion of one gene copy diminished *CHT3* transcript levels by about 90% in both backgrounds. In strain 124A, showing two-fold higher *CHT3* expression than SC5314, loss of *CHT3* was compensated by upregulation of *CHT2*. Infection studies with *cht3*Δ mutants in strain 124A showed that *CHT3* deletion led to attenuated virulence. Histological analysis of infected kidneys showed that *CHT3* deletion did not affect the morphology of *C. albicans* cells during infection, but it appeared to delay activation of macrophages for efficient yeast killing. In conclusion, this study demonstrated that Cht3 activity is required for normal cell separation during yeast growth, cell surface organization, and full virulence of *C. albicans in vivo*. Its importance for virulence aligns with the earlier observed potential of Cht3 as vaccine candidate and warrants further studies to elucidate the mechanisms underlying its role in virulence and interaction with the host immune system.

## 1 Introduction

The *Candida albicans* genome harbors four distinct chitinase encoding genes: *CHT1 - 4*. Cht1, 2, and 3 are homologous to *Saccharomyces cerevisiae* Cts1 (McCreath et al., 1995; Selvaggini et al., 2004), whereas Cht4 has closer homology to *S. cerevisiae* Cts2. Analysis of *CHT1*, *2* and *3* deletion mutants in auxotrophic parental backgrounds indicated that, similar to ScCts1, Cht3 is essential for proper cell separation during growth and division of *C. albicans* yeast cells (Dünkler et al., 2005; Selvaggini et al., 2004). In contrast, deletion of *CHT1* and *CHT2* was observed to have only limited effects under the tested conditions and did not affect cell separation (Dünkler et al., 2005; Iranzo et al., 2002). Studies on *CHT4* deletion mutants have not been reported yet but its heterologous expression was shown to complement sporulation defects of *cht2* mutants in *Ashbya gossypii* (Dünkler et al., 2008).

Given its involvement in cell division, *CHT3* displays higher expression levels in cultures containing unicellular yeasts compared to those with hyphal growth. The same was observed for *CHT2* while expression of *CHT1* could not be detected in either condition (McCreath et al., 1995). Supporting the notion that Cht3 is the predominant chitinase in *C. albicans*, deletion of *CHT3* led to drastically decreased chitinase activity in both cell-associated as well as culture supernatant fractions. *CHT1* deletion did not result in diminished chitinase activity, while for *cht2Δ* mutants this was inconclusive as only a slight decrease in yeast cell-associated activity was reported in one of the papers (Dünkler et al., 2005; Selvaggini et al., 2004). Cht2 has been found to be covalently attached to the cell wall (De Groot et al., 2004; Iranzo et al., 2002) and specifies a predicted C-terminal glycosylphosphatidylinositol (GPI) anchoring peptide (De Groot et al., 2003). Cht1, 3 and 4 lack GPI anchoring sequences and are therefore likely to be secreted, consistent with the diminished chitinase activity in the growth medium of *cht3*Δ mutants (Dünkler et al., 2005).

Expression of *CHT3* peaks during the M/G_1_ phase of the cell cycle (Côte et al., 2009). This temporal peak in expression is linked to the role of Cht3 in cell division, where it participates in the cleavage of chitin at the septum formed during this process. Thus, deletion of *CHT3* leads to defects in cell separation, resulting in the formation of cell chains or agglomerates with chitin accumulation at the budding site, while hyphal growth remains unaffected by the Cht3 knockout (Dünkler et al., 2005; Klis and Brul, 2015). The *CHT3* expression peak during the M/G_1_ phase coincides with the activation of other genes implicated in cell separation such as *ENG1*, *SCW11*, and *DSE1*, which encode an endo-β-1,3-glucanase, a β-1,3-glucan-modifying enzyme, and a daughter cell-specific protein of unknown function, respectively, all controlled by the transcriptional regulator Ace2 (Mulhern et al., 2006). Also in line with their implication in cell separation, proteomic studies showed elevated levels of Cht3, Eng1, and Scw11 in the culture solution of unicellular budding yeasts compared to hyphal cells (Klis and Brul, 2015; Sorgo et al., 2010).

In our previous studies, Cht3 emerged as an antigen of interest when a dithiothreitol (DTT) extract from intact cells encapsulated in dioctadecyldimethylammonium bromide:monoolein (DODAB: MO) liposomes was utilized for immunizing mice (Carneiro et al., 2015, 2016). In further studies, immunization with recombinant Cht3 combined with secreted aspartyl proteinase 2 (Sap2), encapsulated in the same liposomes, led to a significant protection of mice against lethal systemic candidiasis and stimulated high antibody production (Costa-Barbosa et al., 2025). Interestingly, a formulation containing only the recombinant Cht3 in the liposomes induced the production of anti-Cht3 antibodies capable of opsonizing yeast cells at the mother-daughter scars. These findings confirmed the active presence of Cht3 at these sites in the cell wall, consistent with its implication in cell separation (Costa-Barbosa et al., 2023).

We aim to deepen our understanding of the role of Cht3 in *C. albicans* cell wall organization and virulence and correlating it with the protective effects observed previously. The recombinant protein, purified and applied as a vaccine candidate in our previous studies, originated from reference strain SC5314 and, notably, demonstrated protective activity against a clinical isolate (strain 124A). The *cht* mutants generated in earlier studies utilized auxotrophic selection markers to delete chitinase genes in a parental strain with deletions in *URA3*, *HIS1*, and *ARG4* or applied the Ura-blaster system (Dünkler et al., 2005; Selvaggini et al., 2004). These techniques have limitations, as the imposed genetic alterations and procedures can interfere with proper marker gene expression, potentially affecting cellular physiology, which might result in misleading outcomes of virulence studies (Correia et al., 2010). Therefore, in this study, we generated and phenotypically characterized *cht3*Δ deletion mutants in wild-type (WT) backgrounds, clinical isolate 124A and reference strain SC5314, utilizing the *SAT1* flipper system (Reuß et al., 2004).

## 2 Materials and methods

### 2.1 Strains and growth conditions

Strains used in this study are listed in **Supplementary Table S1**. Unless stated otherwise, yeast strains were routinely (pre-)cultured in YPD medium (1% (w/v) yeast extract, 2% (w/v) peptone, 2% (w/v) glucose), supplemented with 2% (w/v) agar for solid media when required, and incubated at 37°C.

### 2.2 *CHT3* gene deletion

*CHT3* (GenBank Number: XM_714255.1) deletion was carried out using the *SAT1* flipper system (Reuß et al., 2004) combined with CRISPR RNA-Cas9 ribonucleoprotein complexes (RNPs) (Grahl et al., 2017) to increase the efficiency of integration into the correct locus, following procedures described previously (Reithofer et al., 2021). In brief, *CHT3* flanking regions of about 500 bp were amplified (see **Supplementary Table S2** for primer sequences) and cloned into plasmid pSFS2. The sequence-verified deletion cassette was mixed with a CRISPR guide (**Supplementary Table S2**) and Cas9 protein (IDT, Leuven) and transformed into *C. albicans* cells by electroporation (Grahl et al., 2017). Deletion mutants were selected following protocols described by Reuß et al (Reuß et al., 2004). Finally, PCR validation of mutants and excision of the cassette was performed as shown in **Supplementary Figure S1**.

### 2.3 Growth kinetics

To assess growth kinetics, precultured cells of *cht3Δ* mutants and the corresponding parental strains were diluted to an initial OD_600_ of 0.1 in fresh YPD medium and incubated in 96-well plates at 30°C and 37°C. The increase in OD_600_ was recorded hourly following a five-seconds agitation step using a SpectraMax 340 microplate reader (Molecular Devices). Data represent the mean of three independent biological replicates measured in triplicate.

### 2.4 Cell separation assays

Effects of deleting the *CHT3* gene on cell separation were assessed by flow cytometry (FC) with cultures grown at 37°C to logarithmic (OD_600_ = 1 - 2) or stationary phase. Harvested cells were fixed with 4% paraformaldehyde for 30 min, washed with PBS, and analyzed on a MACSQuant^®^ Analyzer 10 (Miltenyi Biotec). For each sample, 20,000 events were recorded of which single-cell events were identified through a two-step gating strategy (FSC-A vs. FSC-W followed by SSC-A vs. SSC-W). Gates were defined independently for each genetic background as the two WT parental strains showed slightly different morphological behavior in this experimental procedure. Data were analyzed using FlowJo™ v10.10.0 software (BD Life Sciences). The proportion of single-cell events was calculated relative to the total cellular population, with reductions being interpreted as impaired cell separation. Representative images of the same cell cultures were acquired using a Leica DM1000 brightfield microscope equipped with an MC170 HD digital camera to validate the FC data.

### 2.5 Sedimentation

Stationary phase cultures grown at 37 °C were transferred to 15 cm long glass tubes and allowed to sediment at room temperature (RT). Sedimentation was monitored over time by measuring the OD_600_ of 20 μL samples collected 2 cm below the liquid surface.

### 2.6 Biofilm formation

Biofilm formation assays were performed in YPD and Roswell Park Memorial Institute (RPMI)-1640 medium buffered to pH 7.0 with 0.165 M morpholinopropanesulfonic acid (MOPS). Cells from overnight precultures were diluted to an OD_600_ of 0.5 in fresh YPD or RPMI - 1640 and incubated in 96-well plates for 24 h at 37°C without shaking. Biofilm mass was quantified using the Crystal violet (CV) assay (Reithofer et al., 2021), while metabolic activity was assessed via the XTT assay (Pierce et al., 2010) as previously described. Data shown are the mean of four independent biological replicates per strain, each measured with six technical replicates.

### 2.7 Biochemical determination of the chitin and protein cell wall content

Cell wall isolation from cells grown to logarithmic phase at 37°C was performed as previously described (De Groot et al., 2004). Chitin and protein content in the samples were determined using colorimetric assays according to (Kapteyn et al., 2001) and values were expressed as a percentage of dry cell wall weight.

### 2.8 Quantitative flow cytometry analysis of chitin and confocal microscopy

Yeast cultures were prepared for FC analysis as described above. Chitin in fixed cells (2 × 10^7^) was stained with wheat germ agglutinin conjugated to fluorescein isothiocyanate (WGA-FITC; 100 μg/mL, Sigma-Aldrich) for 1 h, or for 5 min with Calcofluor white (CFW, 25 μg/mL, Glentham Life Science), both at 37°C. Following staining, cells were washed three times with PBS and fluorescence intensity was measured by FC, acquiring 50,000 events per sample. For each strain, an autofluorescence threshold was established based on the median fluorescence intensity (MFI) of the corresponding unstained control population.

Confocal microscopy was performed using the same fluorescent dyes. For dual labeling of chitin and mannan, cells were first incubated for 15 min with WGA-FITC, after which Concanavalin A conjugated to tetramethylrhodamine (ConA-Rho; 100 μg/mL, Molecular Probes) was added and incubation was continued for another 45 min. Cells were washed with PBS and mounted for imaging. Confocal acquisition was performed using a Zeiss LSM 710 Laser Scanning Microscope equipped with a Plan-Apochromat 63×/1.40 oil immersion objective (DIC M27). Images were acquired at 8-bit depth and 1024 × 1024 resolution and processed using ZEN software (version 3.8.99.00000).

### 2.9 Response to cell surface-perturbing agents

Sensitivity to cell surface-perturbing agents was assessed by spotting 4 µL of yeast precultures diluted to an OD_600_ of 1.0 and four serial ten-fold dilutions thereof onto YPD agar supplemented with Congo red (CR), CFW, caffeine (10 mM), caspofungin (90 ng/mL) or SDS (0.035% (w/v)). CR and CFW concentrations were adjusted according to the sensitivity of the parental strain background: 100 μg/mL CFW and 75 μg/mL CR for SC5314, and 25 μg/mL of each compound for strain 124A. Plates were incubated at 37°C and growth was documented by photography after 24 and 48 h. At least two individual experiments were performed to confirm the observed phenotypes, each including two biological replicates.

CFW susceptibility was also quantified using broth microdilution assays in 96-well plates, adapted from EUCAST guidelines (Guinea et al., 2023; Reithofer et al., 2021). Briefly, cell suspensions adjusted to an OD_600_ of 0.01 were mixed 1:1 with CFW serial dilutions in YPD and incubated at 37°C for 24 h with continuous shaking. The minimal inhibitory concentration (MIC_50_) values were determined based on optical density measurements.

### 2.10 Zymolyase sensitivity

Logarithmic phase cells were collected, washed and adjusted to an OD_600_ of 2.0 in 10 mM Tris-HCl buffer (pH 7.4) containing 0.25% β-mercaptoethanol, followed by a 1 h incubation at RT. Cell suspensions and Zymolyase-20T (Amsbio) were combined in 96-well plates to achieve a final enzyme concentration of 1 U/mL. Lysis was monitored at 37°C, with OD_600_ readings taken at 1-min intervals following brief agitation. Curves represent the mean of three independent experiments, each including two biological replicates analyzed in triplicate.

### 2.11 Quantitative real-time PCR analysis of *CHT* gene expression

Parental strains SC5314 and 124A, along with their respective *cht3*Δ mutants, were cultured to exponential or stationary phase at 37°C. Total RNA was extracted following mechanical lysis with a Fastprep-24 instrument (MP Biomedicals) using TRIzol reagent (Thermo Fisher Scientific) and standard protocols. One microgram of RNA was reverse-transcribed into cDNA with the High-Capacity RNA-to-cDNA kit (Applied Biosystems). qPCR was carried out with a 7500 Fast Real-Time PCR system (Applied Biosystems) using 4 μL of 20-fold diluted cDNA and Fast SYBR Green qPCR Master Mix (Thermo Fisher Scientific) in a 10 μL reaction volume. Primer sequences are listed in **Supplementary Table S2**. Primer specificity and amplification efficiency for all targets, including the reference genes *ACT1* and *RIP1*, were validated by standard curve analysis and by agarose gel electrophoresis. Ct values were corrected to *ACT1* expression and normalized against *RIP1* expression levels.

### 2.12 *In vivo* virulence in the mouse model of disseminated candidiasis

#### 2.12.1 Mouse husbandry

Female BALB/c mice, eight to ten weeks old, were purchased from Charles River (Barcelona, Spain) and kept under specific pathogen-free conditions at the Institute for Research and Innovation in Health (i3S) (Porto, Portugal). All procedures involving mice were performed according to the European Convention for the Protection of Vertebrate Animals used for Experimental and Other Scientific Purposes (ETS 123), the 2010/63/EU directive, and Portuguese rules (DL 113/2013). Procedures were approved by the i3S institutional board responsible for animal welfare (ORBEA) and authorization to perform the experiments was issued by the competent national authority (Direção Geral de Alimentação e Veterinária) with the reference number 014036/2019-07-24.i3S.

#### 2.12.2 Infection procedures and kidney collection

Mice were infected with WT (n = 6), heterozygous, and homozygous mutants (n = 8 each) in the 124A background. Yeast precultures were grown on Winge agar (0.3% (w/v) yeast extract, 0.2% (w/v) glucose, 2% (w/v) agar) at 30 °C for 24 h. Colonies were restreaked onto fresh Winge plates and incubated for an additional 14 h. Cells were then collected, washed, and resuspended in PBS to a final concentration of 1 × 10^6^ cells/mL, and 100 µL were injected intravenously in the lateral tail vein. This experiment was independently repeated twice using the same number of animals per group, as described above. Progression of hematogenously disseminated candidiasis was evaluated in accordance with international guidelines, by monitoring survival, clinical behavior, appearance, body weight, temperature, hydration status, and respiratory patterns. These parameters were used to calculate animal welfare scores. Mice were euthanized by cervical dislocation upon reaching humane endpoints or at the conclusion of the experimental period.

Kidneys from euthanized mice were collected and fixed in 3.7% buffered formalin for histological analysis. Paraffin-embedded sections were stained with periodic acid–Schiff (PAS) reagent and counterstained with hematoxylin–eosin (HE) to visualize fungal morphology. Imaging was performed using a Leica DM500 microscope equipped with a Leica ICC50 W camera and LAS V4.12 software (Leica Microsystems).

### 2.13 *In vivo* virulence in *Galleria mellonella*

In addition to the murine model, virulence was also assessed using the invertebrate *G. mellonella* infection model with WT and mutants in both genetic backgrounds, following a previously described protocol (Alvarado et al., 2024).

### 2.14 Macrophage killing assay

The murine macrophage cell line J774A.1 was maintained in high-glucose (4.5 g/L) Dulbecco’s Modified Eagle Medium (DMEM; Gibco), supplemented with 10% heat-inactivated fetal bovine serum (FBS; Gibco), 2 mM L-glutamine, 1 mM sodium pyruvate, and 25 mM HEPES, under standard conditions (37°C, 5% CO_2_, humidified atmosphere) in tissue culture flasks (Nunc). Adherent macrophages were detached by gentle scraping and counted using Trypan blue exclusion (Sigma-Aldrich). Cells were adjusted to 1 × 10^5^ cells/mL in complete DMEM, and 100 μL were seeded per well in 96-well plates, followed by overnight incubation. Spent medium was removed and replaced with 200 μL of yeast cell suspension, previously subcultured overnight in YPD and adjusted to 2.5 × 10^6^ cells/mL in DMEM. The plates were then incubated for an additional 4 h at 37°C. Finally, macrophages were lysed with 10% saponin (Sigma-Aldrich), and colony-forming units (CFUs) were determined by plating on YPD agar. Control wells included macrophages alone and yeast alone. Data represent the mean of three independent biological replicates, each measured in triplicate. Imaging of parallel 20 h yeast–macrophage co-incubations, including assays using macrophages pre-activated overnight by exposure to heat-killed *Candida* cells, was conducted with a THUNDER Ready DMi8 inverted fluorescence microscope (Leica Microsystems).

### 2.15 Statistical analysis

Unless mentioned otherwise, all phenotypic assays were performed with four biological replicates, measured in triplicate. Statistical analyses were conducted using GraphPad Prism 9 (GraphPad Software). For datasets with normal distribution, one-way ANOVA followed by Tukey´s *post hoc* test was applied to determine significance. Survival data were analyzed using the Kaplan-Meier method and compared with the Mantel-Cox log-rank test. Statistical significance is indicated as follows: *p *<* 0.05; **p *<* 0.01; ***p *<* 0.001; ****p < 0.0001.

## 3 Results

### 3.1 *CHT3* deletion in WT backgrounds affects cell separation

Given its potential as a promising vaccine candidate, this study aims to further elucidate the role of the chitinase Cht3 in *C. albicans*, particularly in cell surface remodeling during growth and infection. Previous studies have generated *cht3*Δ mutants using strains with auxotrophic markers or the Ura-blaster system (Dünkler et al., 2005; Selvaggini et al., 2004). As the genetic modifications in such parental strains might affect phenotypic studies, here we generated and studied mutants in - two different - WT backgrounds: reference strain SC5314 (clade 1; Odds et al., 2004) and clinical isolate 124A. The latter is a clade 16 strain isolate from an immunocompromised patient with invasive candidiasis (Sampaio et al., 2010) against which prior vaccination with a Cht3-containing DTT cell extract from SC5314 provided protection (Carneiro et al., 2016; Costa-Barbosa et al., 2023).

Implementing the CRISPR-Cas9 technique during transformation enabled the generation of homozygous *cht3*Δ mutants through two independent strategies: either by simultaneous deletion of both alleles in a single transformation event, or by sequential deletion of the two alleles (**Supplementary Table S1**). In all phenotypic assays, the independent mutants showed identical phenotypic behavior.

**Figure 1:**
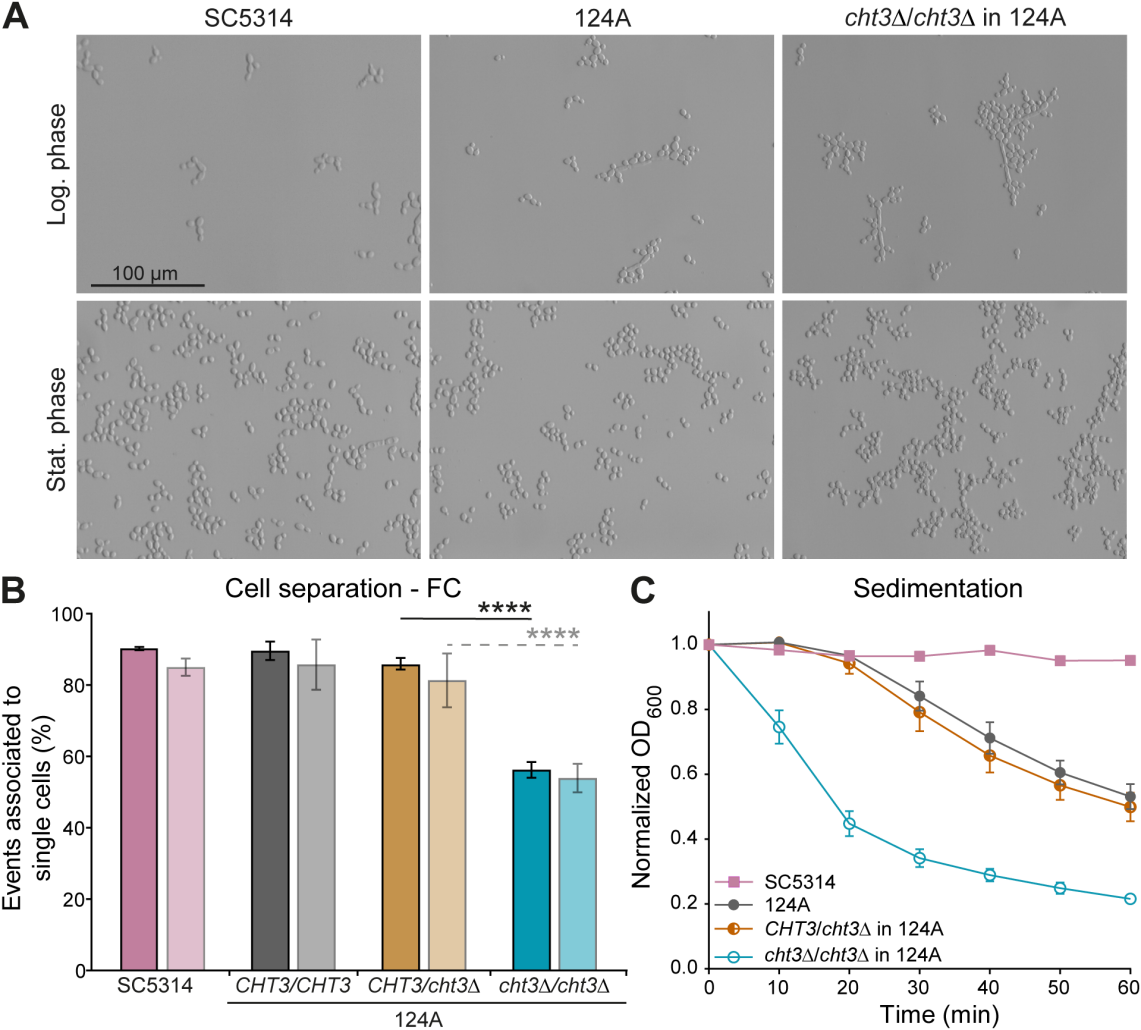
Deletion of *C*. *albicans CHT3* impairs cell separation. **(A)** Cell morphology at 37°C as shown by optical microscopy. **(B)** Cell separation defects in logarithmic and stationary phases (dark and light bars, respectively) quantified by flow cytometry (FC) as detailed in Materials and Methods. **(C)** Sedimentation assays. Data are the mean ± standard deviation (SD) of four biological replicates measured in triplicate. Statistical differences were determined by one-way ANOVA with Tukey’s *post hoc* test. ****p < 0.0001.

Deleting *CHT3* in both strain backgrounds had no measurable impact on their fitness at 30°C or 37°C (**Supplementary Table S3**). However, microscopical analysis showed a clear defect in cell separation at both temperatures in both backgrounds in the homozygous but not the heterozygous mutants (**Figure 1A**; **Supplementary Figure S2A**; data at 30°C not shown). Cell separation at 37°C was further studied quantitatively by FC following a washing step in PBS, confirming the microscopic observations (**Figure 1B**; **Supplementary Figure S2B**). Results of complementary sedimentation assays were consistent with the cell separation phenotype, and further highlighted differences between the genetic backgrounds. The 124A strain sedimented significantly faster than the SC5314 WT at the later timepoints (**Figure 1C**; **Supplementary Figure S2C**), suggesting the formation of larger or more cohesive cellular aggregates.

Such cellular aggregation may, in turn, influence biofilm formation, thereby potentially linking alterations in cell surface architecture to pathogenic potential. Both strains exhibit low biofilm formation in YPD medium. In contrast, reference strain SC5314 but not clinical isolate 124A forms significantly more biofilm mass in RPMI - 1640 medium (**Supplementary Figure S3A**), however, this was not altered by deletion of *CHT3* (not shown). These results were corroborated by the biofilm metabolic activity (XTT) assay (**Supplementary Figure S3B**).

### 3.2 Cht3 activity affects the cell wall organization in surface-exposed chitin

**Figure 2:**
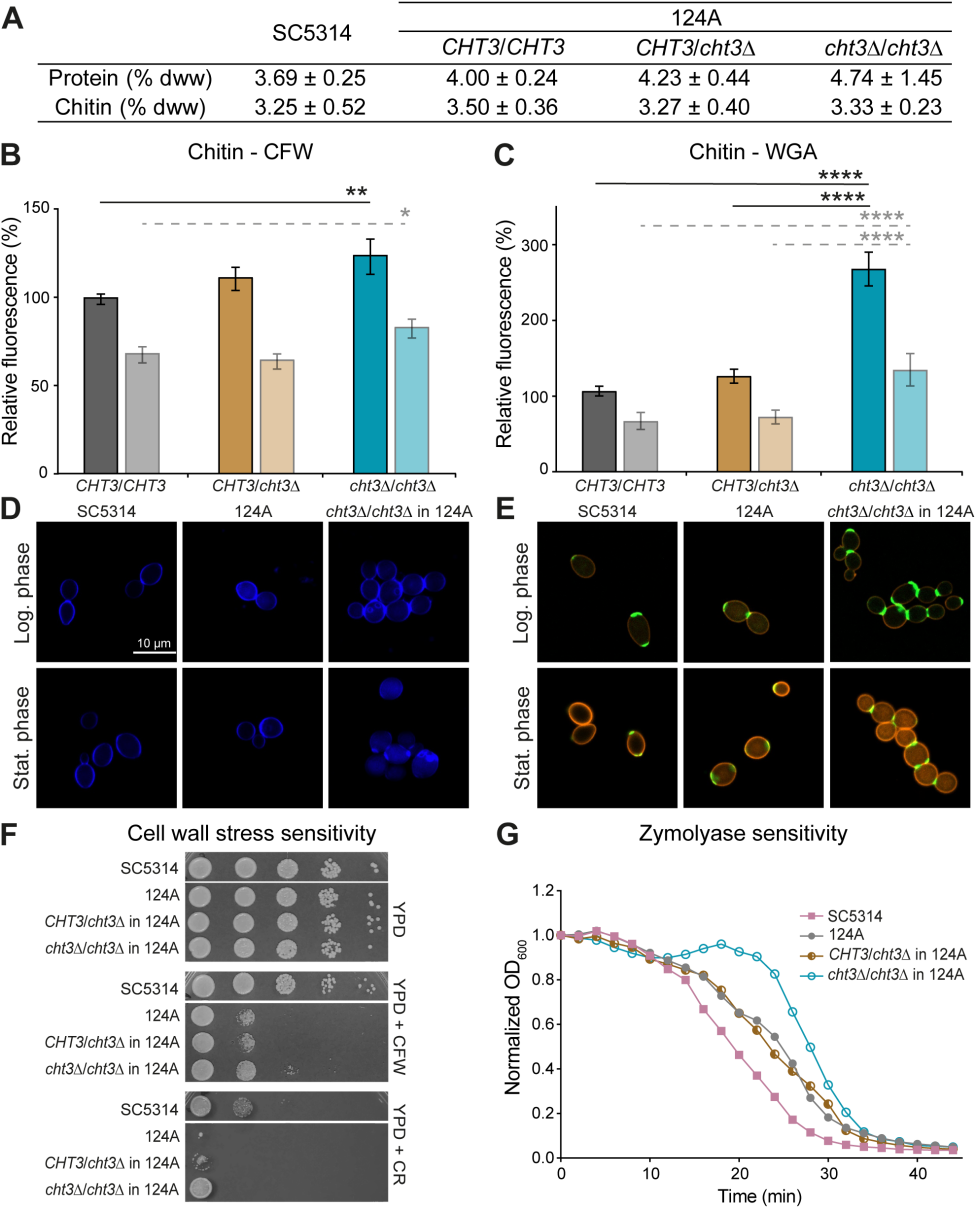
*CHT3* deletion leads to changes in cell wall organization. **(A)** Biochemical determination of total cell wall chitin and protein content, expressed as percentage of dry cell wall weight (% dww) ± SD. **(B, C)** Flow cytometry analysis of **(B)** Calcofluor white (CFW) and **(C)** wheat germ agglutinin (WGA) chitin binding measured in logarithmic (dark bars) and stationary (light bars) phases. Median fluorescence intensities (MFI) were normalized to the MFI of the reference strain SC5314 in logarithmic phase. **(D, E)** Representative fluorescence microscopy images showing chitin localization using **(D)** CFW and **(E)** WGA-FITC. WGA-FITC staining was combined with Concanavalin A-rhodamine to label cell wall mannan. **(F)** Cell wall stress resistance on YPD agar supplemented with CFW (25 μg/mL) or Congo Red (CR; 25 μg/mL). Photographs were taken after 24 h of growth at 37°C. **(G)** Zymolyase sensitivity. Data shown in **(A-C)** are the mean ± SD of four biological replicates measured in triplicate. Statistical differences were assessed using one-way ANOVA and Tukey’s *post hoc* test. Curves in **(G)** represent the mean of three independent experiments, each including two biological replicates analyzed in triplicate. *p *<* 0.05; **p *<* 0.01; ****p < 0.0001.

As Cht3 plays a key role in mother-daughter cell separation, we studied the impact of *CHT3* deletion on cell wall organization (**Figure 2**; **Supplementary Figure S4**). Total chitin content was first quantified biochemically. This assay revealed no significant differences between WT strains and *cht3*Δ mutants. Similarly, no variation in protein content was detected in the same samples (**Figure 2A**; **Supplementary Figure S4A**).

In addition, quantitative FC analysis was performed using CFW and WGA-FITC, which are known to bind chitin polymers and exposed *N*-acetylglucosamine residues, respectively (Chaffin et al., 1998; Mora-Montes et al., 2011). During logarithmic growth, homozygous mutants showed a 24 - 25% increase in CFW binding in both genetic backgrounds. Consistent with the absence of cell separation defects, heterozygous mutants did not show increased CFW binding. In the stationary phase, when cell division is ceased, no or almost no increase in CFW binding was observed, with only a marginal increase in the homozygous mutants in strain 124A (**Figure 2B**; **Supplementary Figure S4B**). Remarkably, with respect to WGA binding during logarithmic growth, the heterozygous mutants in SC5314 already showed a marked (almost two-fold) increase, whereas in the 124A background only the homozygous mutants showed (2.5-fold) increased binding. The latter tendency was also the case during the stationary phase in both backgrounds (**Figure 2C**; **Supplementary Figure S4C**). More intense staining in confocal fluorescent microscopy images (**Figures 2D, E**; **Supplementary Figures S4D, E**) corroborates these data. Thus, rather than affecting the total cell wall chitin, these data point to differences in surface-exposed chitin molecules because of the presence or absence of Cht3 chitinase activity.

To assess alterations in cell wall homeostasis, the sensitivity of WT and *cht3*Δ mutants to various cell surface stressors was evaluated (**Figure 2F**; **Supplementary Figure S4F**). Drop assays showed that the clinical strain (124A) is more sensitive to CFW and CR than the reference strain (SC5314). In both genetic backgrounds, homozygous mutants exhibited increased resistance to these stresses compared to both parental and heterozygous strains. However, this effect was less pronounced in the clinical isolate under CR exposure, likely due to its inherent high sensitivity. CFW spot assay results were confirmed by microdilution assays (data not shown), which demonstrated a four-fold higher resistance in SC5314 compared to 124A. Further, homozygous deletion of *CHT3* led to a four-fold increased resistance in SC5314, but not in 124A background. In contrast, spot assays with other stressors such as caffeine (a cell wall integrity pathway inhibitor), SDS (a membrane-disrupting detergent), or caspofungin (a β-1,3-glucan synthase inhibitor), did not show differences between WT and mutants in either background (data not shown). To further evaluate cell wall integrity, sensitivity to Zymolyase, a β-1,3-glucanase preparation, was tested in cells grown to logarithmic phase at 37°C (**Figure 2G**; **Supplementary Figure S4G**). The results indicated that the SC5314 strain is slightly more sensitive than the clinical strain. In both genetic backgrounds, the homozygous mutants exhibited a distinct resistance “blob”, which was more pronounced in the 124A strain. Although not clear, this resistance could be related to the separation defect observed in the homozygous mutants.

### 3.3 High expression of *CHT3* in strain 124A but not in SC5314 leads to compensatory upregulation of *CHT2*

**Figure 3:**
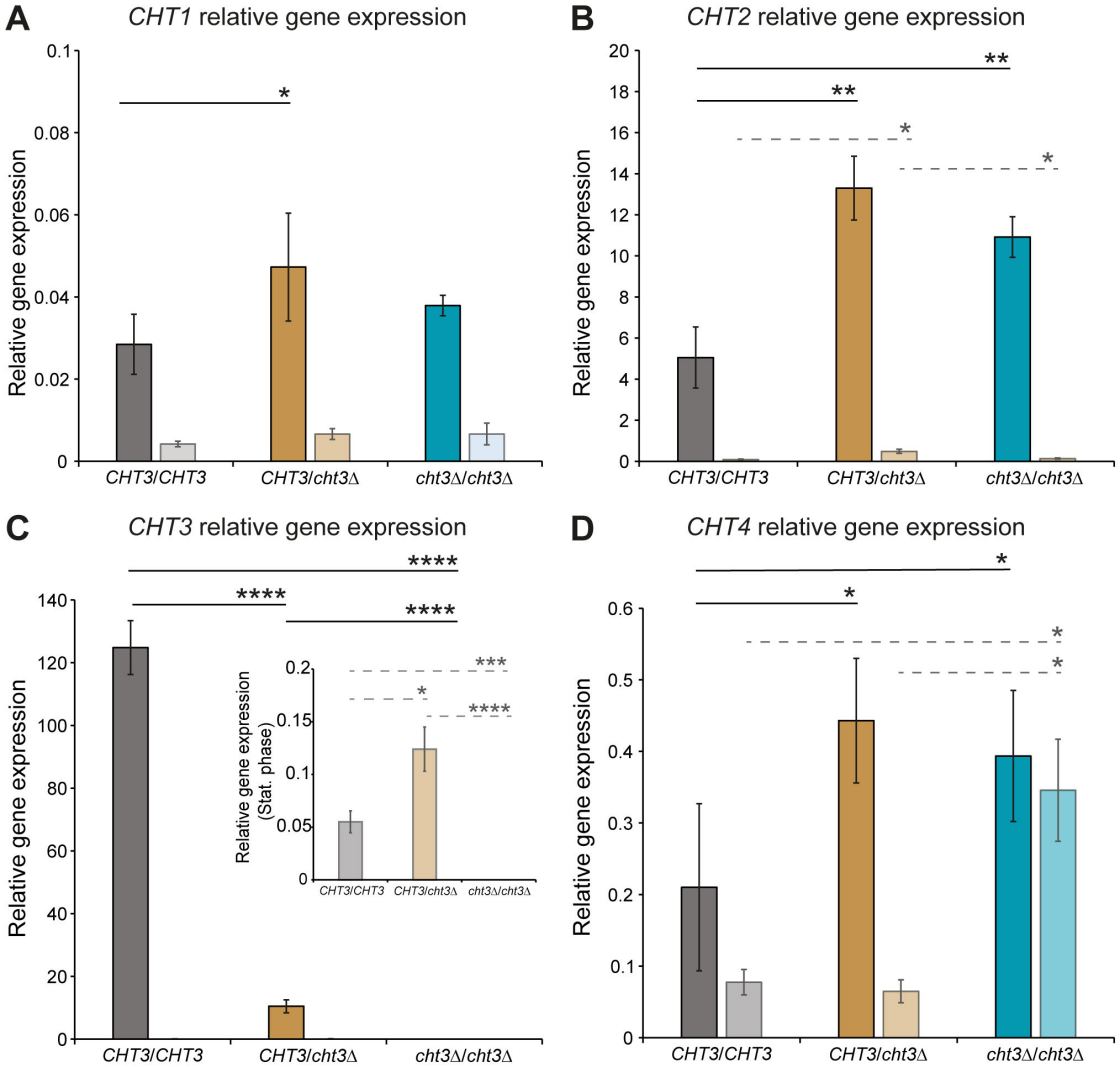
qPCR gene expression analysis of *CHT1–4* genes in *C. albicans* strain 124A. **(A)** *CHT1*, **(B)** *CHT2*, **(C)** *CHT3*, and **(D)** *CHT4*. WT and *cht3*Δ deletion mutants were grown to logarithmic (dark bars) and stationary (light bars) phases. Data were normalized to *ACT1* expression and plotted in comparison to *RIP1* transcript levels. Due to their low levels, *CHT3* transcript levels in stationary phase are also displayed in an inset graph with adjusted y-axis. Data shown are the mean ± SD of two biological replicates measured in triplicate. Statistical analysis was performed by one-way ANOVA followed by Tukey´s *post hoc* test. *p *<* 0.05; **p *<* 0.01; ***p *<* 0.001; ****p < 0.0001.

Potential compensatory transcriptional responses to *CHT3* deletion were measured by qPCR of the four chitinase genes in cells grown to exponential and stationary phases (**Figure 3**; **Supplementary Figures S5, S6**). Consistent with their proposed role in cell separation during growth, *CHT1*, *CHT2*, and *CHT3* showed highest expression during the logarithmic phase, while their transcript levels were much lower in stationary phase. A similar trend was observed for *CHT4*, a gene linked to sporulation (Dünkler et al., 2008), although the difference between the growth phases was less pronounced. Among the four chitinase genes analyzed, *CHT3* exhibited the highest expression levels, followed by *CHT2*. In contrast, *CHT1*, and to a lesser extent *CHT4*, were minimally expressed in both genetic backgrounds, with transcript levels barely exceeding background detection thresholds. Notably, loss of only one *CHT3* allele led to a marked reduction in *CHT3* expression during logarithmic growth in both SC5314 and 124A strains, indicating a strong dependence on gene dosage. Interestingly, deletion of *CHT3* was accompanied by a two- to three-fold compensatory upregulation of *CHT2* in both heterozygous and homozygous *cht3*Δ mutants in the 124A background, a response that was not observed in SC5314. There, homozygous *CHT3* deletion resulted in a three-fold decrease in *CHT2* expression. This strain-dependent difference was further reflected in the global expression profiles of the chitinase genes during logarithmic growth. While they generally showed higher expression in SC5314, *CHT3* showed higher transcript levels in 124A. The loss of this high *CHT3* expression in 124A might explain the possible compensatory upregulation of *CHT2* in both the heterozygous and the homozygous mutants observed in this background. Finally, the qPCR analysis also validated the *CHT3* gene deletions.

### 3.4 Deletion of *CHT3* decreases *in vivo* virulence of *C. albicans*

As previously described, homozygous *cht3*Δ mutants from both genetic backgrounds show cell separation defects when grown in liquid media. Interestingly, this was not the case when cells were cultured on solid Winge media at 30°C (**Supplementary Figure S7A**), a nutrient-limited medium compared to YPD. This medium was therefore chosen to prepare yeast inocula for infection experiments.

**Figure 4:**
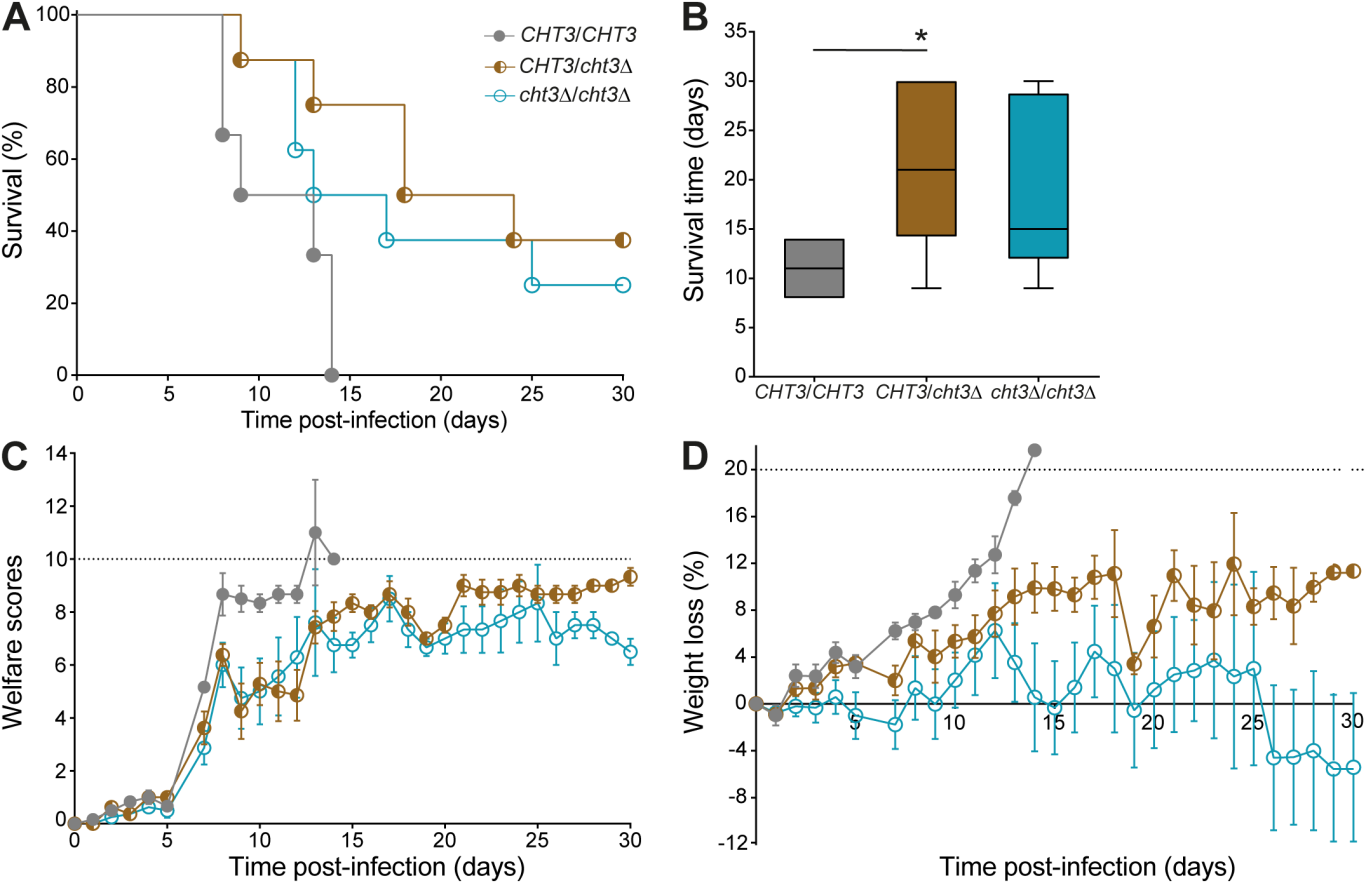
Evaluation of *in vivo* virulence of *cht3*Δ mutants in mice. **(A)** Kaplan-Meier survival curves of mice infected with *C. albicans* 124A WT and corresponding *cht3*Δ mutants. Statistical analysis was performed using the Mantel-Cox log-rank test. **(B)** Median survival times of mice following infection, analyzed by one-way ANOVA followed by Tukey´s *post hoc* test. *p < 0.05. **(C)** Welfare scores and **(D)** weight loss of mice upon infection. Data in **(C, D)** are represented as the mean ± the standard error of the mean.

Mice infected with either homozygous or heterozygous *cht3*Δ mutants in the 124A strain background exhibited better survival and welfare compared to those infected with the WT strain (**Figure 4**). All animals challenged with the WT strain succumbed to infection by day 14, with a median survival time of eleven days. In contrast, median survival times for mice infected with the heterozygous and homozygous mutants were 21 and 15 days, respectively (**Figures 4A, B**). While the difference with WT reached statistical significance for the heterozygous mutant (p = 0.0139), this was not the case for the homozygous mutant (p = 0.0810); though, also no significant differences were observed between the two different mutant strains (p = 0.4556). Welfare scores and weight loss analysis further supported these findings, with both mutant-infected groups showing better outcomes compared to WT-infected mice (**Figures 4C, D**). Animals infected with the homozygous mutant exhibited overall lower weight loss than those infected with the heterozygous mutant. Altogether, these results indicate that deletion of a single *CHT3* allele is sufficient to reduce virulence *in vivo*. In conclusion, *CHT3* deletion reduces mortality and improves the overall health of the infected mice in comparison to the WT strain.

**Figure 5:**
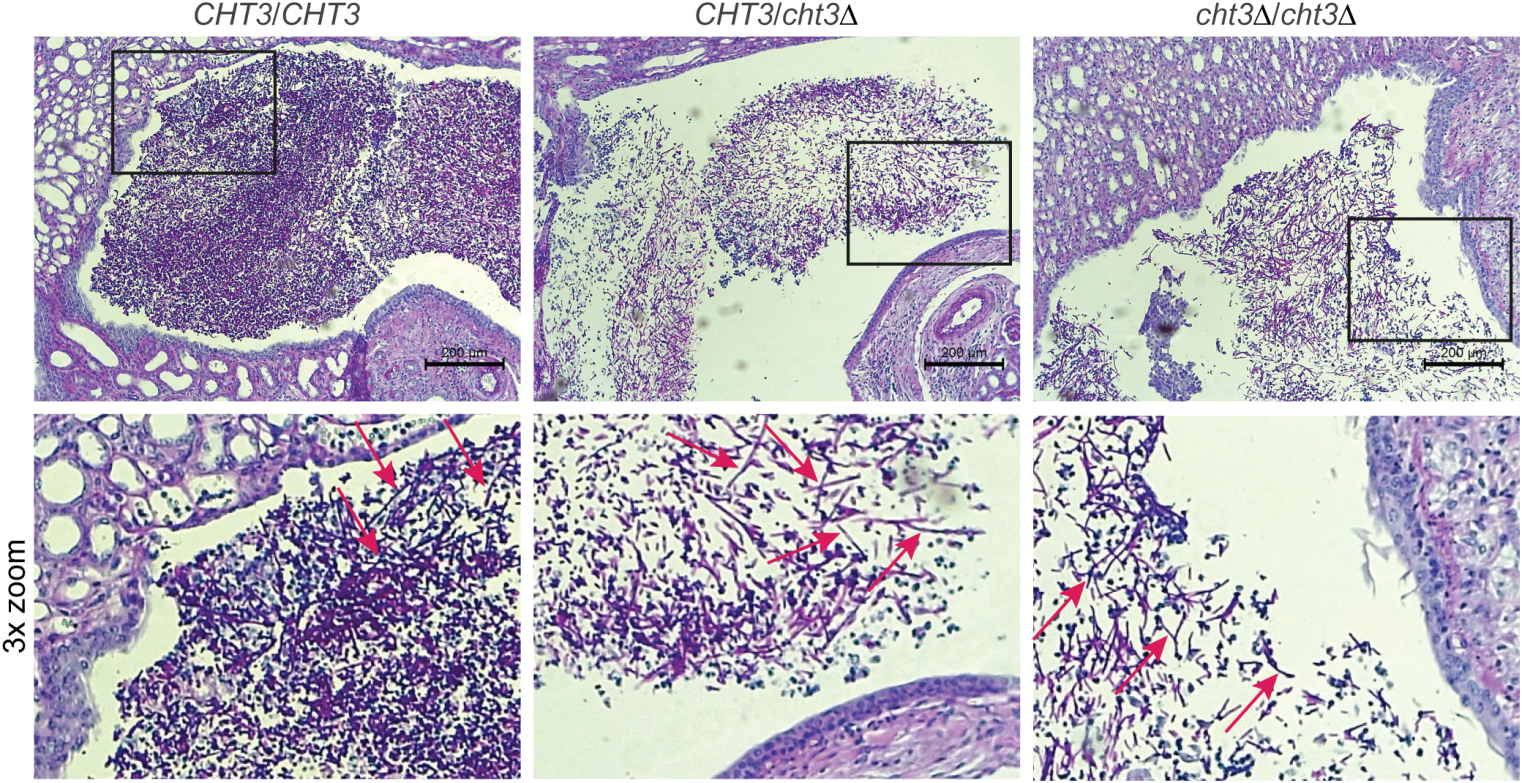
Histological analysis of mouse kidney colonization during infection with *C. albicans*. Representative photomicrographs (top) and zoom-ins of the boxed areas (bottom) from HE/PAS-stained paraffin sections of kidneys collected 13 days post-infection. Shown are samples from mice infected with WT (left), heterozygous (middle), and homozygous (right) *cht3*Δ strains. Arrows indicate *C. albicans* filaments.

*C. albicans* infections in mice often result in kidney colonization (Jawale and Biswas, 2021). Given the lower virulence displayed by the mutant strains, histological analysis of the kidneys of infected animals was carried out (**Figure 5**). In all analyzed mice, filamentous *C. albicans* cells were observed, indicating that deletion of *CHT3* does not impair the capacity of *C. albicans* to filament during the infection process, consistent with *in vitro* filamentation-inducing cultures in liquid Winge (**Supplementary Figure S7B**) and RPMI medium (not shown).

The *in vivo* virulence of *cht3*Δ mutants was also tested using larvae of the wax moth *G. mellonella* as an alternative infection model. In this more primitive host, no significant differences in virulence were observed between the WT strain and the mutants in either genetic background (data not shown).

### 3.5 *CHT3* deletion affects *C. albicans* killing by macrophages

**Figure 6:**
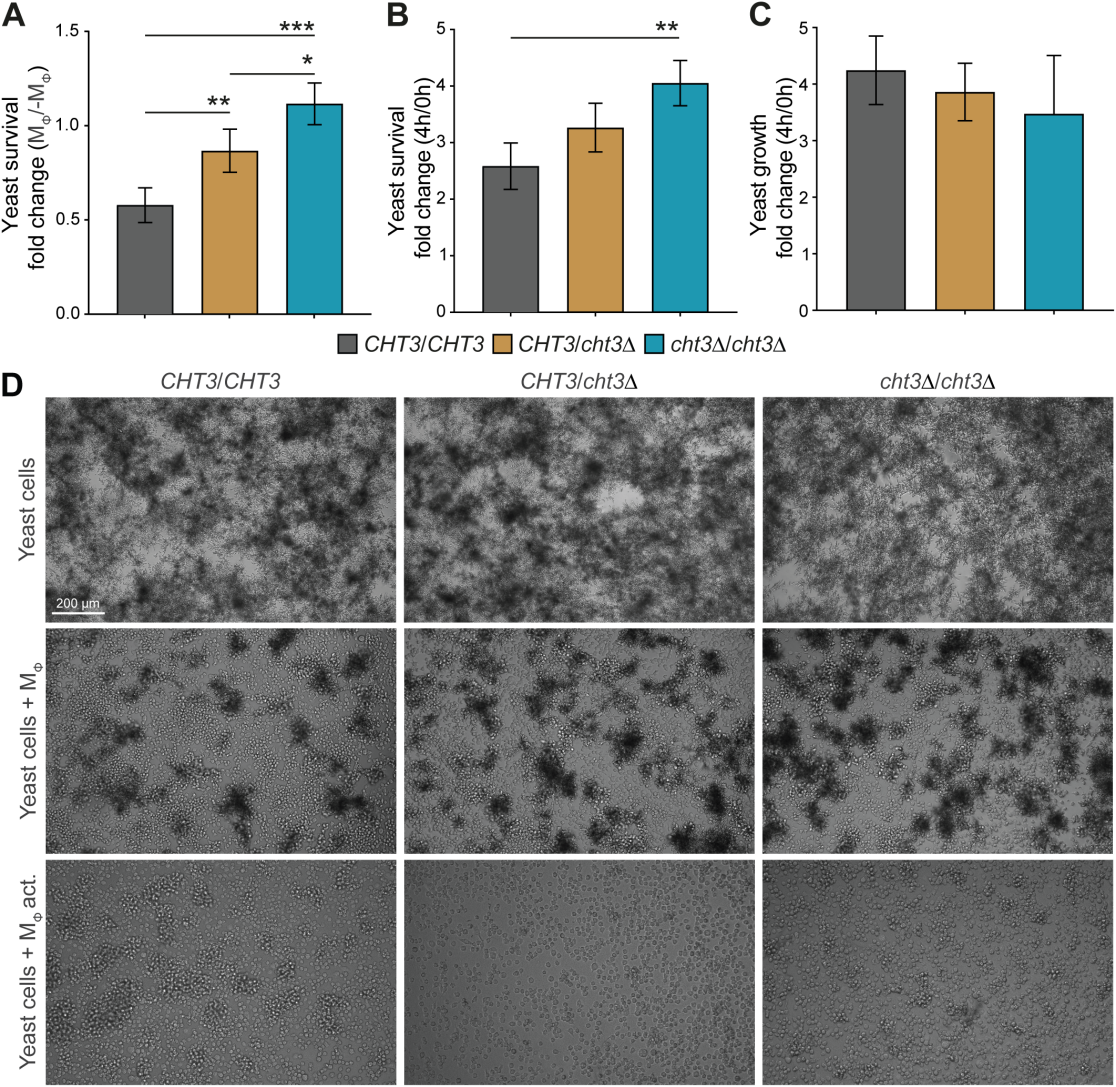
Survival of *C*. *albicans* following macrophage challenge. **(A–C)** WT and mutant strains were challenged with macrophages (M_Φ_) for 4 h Fungal survival was determined based on colony-forming units (CFUs), measured as recovery relative to parallel cultures without macrophages (-M_Φ_) **(A)**, or as fold change in viability between 0 and 4 h post-challenge **(B)**. To assess growth in the absence of immune pressure, CFUs at 4 h were normalized to t = 0 **(C)**. **(D)** Representative images after 20 h of incubation under macrophage-free, non-activated (M_Φ_) or activated conditions (M_Φ_ act.). Data in **(A–C)** represent mean ± SD from four independent experiments. Statistical analyses were performed using one-way ANOVA with Tukey’s *post hoc* test. *p *<* 0.05; **p *<* 0.01; ***p *<* 0.001.

As *cht3*Δ mutants showed reduced virulence *in vivo*, we next explored whether this phenotype could be linked to altered interactions with the host immune system. To this end, the murine macrophage cell line J774A.1 was used as an *in vitro* model to assess fungal survival following co-incubation with immune cells. Survival was quantified by calculating CFU ratios for the WT strain 124A and the corresponding heterozygous and homozygous *cht3Δ* mutants (**Figure 6**). Comparison of CFU ratios after 4 hours of incubation with and without macrophages showed a gene dosage-dependent increase in survival for the mutant strains, indicating enhanced resistance to macrophage-mediated killing (**Figure 6A**). A similar trend was observed when comparing CFU ratios after 4 hours of co-incubation with macrophages relative to the initial time point (**Figure 6B**), suggesting better persistence or proliferation of *cht3Δ* mutants in the presence of immune cells. In the absence of macrophages, no significant differences in growth were observed among the *Candida* strains (**Figure 6C**), indicating that the enhanced survival phenotype is specific to the interaction with macrophages and not due to intrinsic growth differences. To follow on this, incubations with macrophages were extended to 20 h and analyzed by microscopy (**Figure 6D**). As observed at 4 h, all yeast strains exhibited comparable growth in the absence of macrophages. However, during co-incubation, the mutant strains showed increased cell densities compared to the WT strain, especially the homozygous mutant. Moreover, the mutants formed more abundant cellular clumps, also indicative of reduced phagocytosis or killing efficiency. Interestingly, this phenotype was mitigated when macrophages were pre-activated, leading to reduced yeast densities across all strains. These findings suggest that macrophage activation plays an important role in controlling *C. albicans* infection, even in strains with enhanced resistance such as the homozygous *cht3Δ* mutant.

## 4 Discussion

The role of chitinases in *C. albicans* pathogenesis is not precisely known. In earlier studies, of the four chitinases in *C. albicans*, *CHT3* was reported to encode the predominant chitinase and crucial for proper mother-daughter separation during yeast cell division (Dünkler et al., 2005; Selvaggini et al., 2004), findings that are corroborated by our study. However, until now it has not been demonstrated that Cht3 is required for full virulence.

Cht3 is a secreted and cell surface protein that has been identified as a major antigen of interest present in DTT cell extracts during mouse immunization experiments. Since then, it has been employed in the development of a novel vaccine formulation against systemic *C. albicans* infections (Carneiro et al., 2015, 2016; Costa-Barbosa et al., 2023). In this context, immunization with liposomal formulations containing both Cht3 and Sap2 conferred 50% protection against disseminated candidiasis in a murine model (Costa-Barbosa et al., 2025).

Aiming to better understand the role of Cht3 in *C. albicans* cell wall organization and virulence, *CHT3* deletion mutants were generated in two genetically distinct WT strains. The combination of RNP-based CRISPR-Cas9 and the *SAT1* flipper system predominantly yielded heterozygous mutants, with a minority of homozygous deletions obtained in a single transformation event. The initially obtained heterozygous mutants were converted into homozygous through a second round of transformation, resulting in sets of independent mutants exhibiting consistent phenotypic behavior. The use of two distinct genetic backgrounds further adds robustness to our phenotypic analyses. Moreover, as the *SAT1* flipper system does not employ auxotrophic markers, possible influence on virulence phenotypes by ectopic expression of marker genes is being avoided, and ´clean´ mutants are obtained after flipping out the antibiotic resistance marker.

When studying the effect of *CHT3* deletion on yeast growth, homozygous but not heterozygous *cht3*Δ mutants exhibited clear cell separation defects. While this is consistent with earlier studies (Dünkler et al., 2005; Selvaggini et al., 2004) and supports the validity of our mutants, the previous studies were performed at 30°C whereas we consistently performed our studies at the clinically relevant temperature of 37°C. Despite the observed cell separation defect, the growth rate of *C. albicans* cells was not impaired by deleting *CHT3*. The same was true for the filamentation capacity, as observed by histological analysis of infected murine kidneys, *in vitro* culturing, and yeast-macrophage co-incubations. These findings align with previously reported results from *in vitro* hyphal-induction assays (Dünkler et al., 2005). In the latter publication, expression of one *CHT3* allele under control of the regulatable *MAL2*-promoter resulted in the reversion of the cell separation defect when cells were grown in the presence of maltose (Dünkler et al., 2005), confirming the Cht3 dependence of the phenotype. Because of the consistency of the observed phenotypes with the earlier studies, which used different genetic backgrounds and different knockout strategies, we did not further validate our data by incorporating a reconstituted strain. This should be noted as it is considered a relevant point, especially in virulence studies.

Regarding cell wall composition, biochemical determination indicated no differences in total chitin content between WT and mutant strains. However, CFW- and WGA-binding experiments suggested a marked increase in surface-exposed chitin in the mutants. Additionally, *CHT3* deletion led to increased resistance to CFW, CR, and Zymolyase. For CFW, this seems to contradict the somewhat increased sensitivity observed with Ura-blaster mutants (Selvaggini et al., 2004). However, this discrepancy may be attributed to differences in experimental conditions, as the earlier study was conducted at 30°C, whereas our assays were performed at 37°C.

These sensitivity assays also marked architectural differences in the cell walls of the two WT strains used in this study, with SC5314 being more resistant to the cell wall perturbants CFW and CR, but more sensitive to β-1,3-glucan hydrolyzing enzyme preparation Zymolyase. Such differences between the two WT strains, and their different responses to *CHT3* deletion, for instance, in compensatory *CHT2* expression (see below), emphasize the importance of being cautious when extrapolating findings obtained with one genetic background to others.

Significant differences between the two genetic backgrounds used in our study also became apparent when analyzing *CHT* gene expression, in particular, regarding *CHT2* and *CHT3*. The level of *CHT3* expression was approximately two-fold higher in strain 124A than in SC5314. In the clinical isolate background, the loss of this high level of *CHT3* transcription appeared to trigger a compensatory two- to three-fold upregulation of *CHT2* in homozygous as well as heterozygous mutants. This compensatory response was absent in the SC5314 background, where the homozygous mutants in fact showed decreased *CHT2* transcript levels. qPCR analysis also demonstrated that, to sustain mother-daughter cell separation, yeast cells undergoing active cell division showed much higher *CHT* transcript levels compared to cells grown to stationary phase. Furthermore, our results corroborate previous findings that *CHT2* and *CHT3* are the main chitinase-encoding genes in *C. albicans* (Dünkler et al., 2005; McCreath et al., 1995; Selvaggini et al., 2004).

Analysis of mouse survival as well as their welfare scores and weight, revealed that *CHT3* deletion led to attenuated virulence. This reduction might be related to the cell separation defect observed in homozygous *cht3*Δ mutants, as impaired separation might hamper effective dissemination. Therefore, it was somewhat surprising that heterozygous *cht3*Δ mutants, which did not show detected cell separation defects, already exhibited attenuated virulence, and that this phenotype was not exacerbated in homozygous mutants. It is important to stress that *CHT3* expression in the heterozygous mutant was reduced by more than 90%, which seems to explain, at least in part, the lack of sufficient chitinase activity to sustain full virulence. Chitinases have also been linked to pathogenesis in mammalian infections caused by *Paracoccidioides brasiliensis* and in bacterial pathogens, such as *Vibrio cholerae*, *Serratia marcescens*, *Legionella pneumophila*, and *Listeria monocytogenes* (Chaudhuri et al., 2010; DebRoy et al., 2006; Kawada et al., 2008; Pitangui et al., 2021).

Interestingly, in experiments where yeast cells were co-incubated for 4 h with non-activated murine macrophages, *cht3*Δ mutants showed higher survival than the parental strain. However, macrophage pre-activation significantly enhanced antifungal activity, reducing fungal burden across all *CHT3* genotypes. These findings highlight the critical role of the adaptive immune system in orchestrating effective antifungal defenses by activating innate immune cells, particularly macrophages. The enhanced survival of *cht3*Δ mutants only in the presence of non-activated macrophages may stem from impaired immune activity, potentially due to cell separation defects and larger fungal structures, as well as an increased level of surface-exposed chitin, which may interfere with efficient recognition by the immune system (Mora-Montes et al., 2011; Sherrington et al., 2017). The reduced virulence of *cht3*Δ mutants in mice emphasizes the importance of adaptive immunity in priming effective innate responses. The lack of virulence differences in *G. mellonella* may reflect the limitations of this model for studying fungal virulence as this organism possesses a more rudimentary immune system lacking adaptive components. This seems particularly relevant for phenotypes where complex interactions between innate and adaptive immunity are critical to host–pathogen dynamics.

Previous studies have demonstrated that chito-oligomers can induce various types of macrophage activation, depending in part on their size (Da Silva et al., 2009; Fuchs et al., 2018; Wagener et al., 2014). Chito-oligomers with a minimum of six GlcNAc residues can be phagocytosed and activate TLR2 on macrophages, leading to M1 polarization characterized by significant TNF-α production. Smaller oligomers interact with other pattern recognition receptors and induce M2 macrophages, which produce anti-inflammatory cytokines, such as IL-10. When this occurs during the early stages of fungal infection, it suppresses the Th1/Th17 protective immune response (Costa et al., 2013). Paracoccin, a protein with chitinase activity from *P. brasiliensis*, was found to enhance the severity of infections in mice and to hydrolyze cell wall chitin into smaller chito-oligomers. Once released, these chito-oligomers can exert a regulatory role on cytokine production by neighboring macrophages and thus influence the host’s defense mechanisms (Pitangui et al., 2021).

Cht3 can release various chito-oligomers from the chitin/chito-oligomer substrates tested, with larger products being initially produced and these then being broken down to chitobiose as the principal end product, but with chitotriose and the monomer GlcNAc being also observed (Costa-Barbosa et al., 2024). Therefore, the severity of the systemic infection in mice infected with the WT in comparison with the mutants could be partly attributed to a possible immunomodulation by the chito-oligomers produced. However, the exact mechanism responsible for the decrease in virulence in the *C. albicans cht3*Δ mutants remains to be determined.

It is important to note that, while mammals do not synthesize chitin, the presence of mammalian chitinases has been linked to host inflammatory responses against fungi. However, their expression in inflammatory responses in the apparent absence of microbial infection, as seen in diseases like asthma, has made their role puzzling (Kawada et al., 2007; Ober and Chupp, 2009). There are two known human chitinases with chitinolytic activity, chitotriosidase and acidic mammalian chitinase (Lee et al., 2011). The specific targets of these chitinases have not been identified yet, but they might encompass endogenous carbohydrates like heparan sulfate and hyaluronic acid, which share structural similarities with chitin (Lee et al., 2011), or exogenous carbohydrates such as fungal chitin (Vega and Kalkum, 2012), contributing to the presence of chito-oligomers in the medium. Thus, the roles of chitinases in host-fungus interaction, particularly during *C. albicans* infections, is a very relevant topic and still much remains to be unveiled.

## Data Availability

The original contributions presented in the study are included in the article/**Supplementary Material**. Further inquiries can be directed to the corresponding author.
