# Supplementary figures and images for: Loss of CHT3 in Candida albicans wild-type strains increases surface-exposed chitin and affects host-pathogen interaction
Source: Front Cell Infect Microbiol. 2025 Sep 5;15:1654710. doi: 10.3389/fcimb.2025.1654710 (PMC12446338; doi:10.3389/fcimb.2025.1654710)

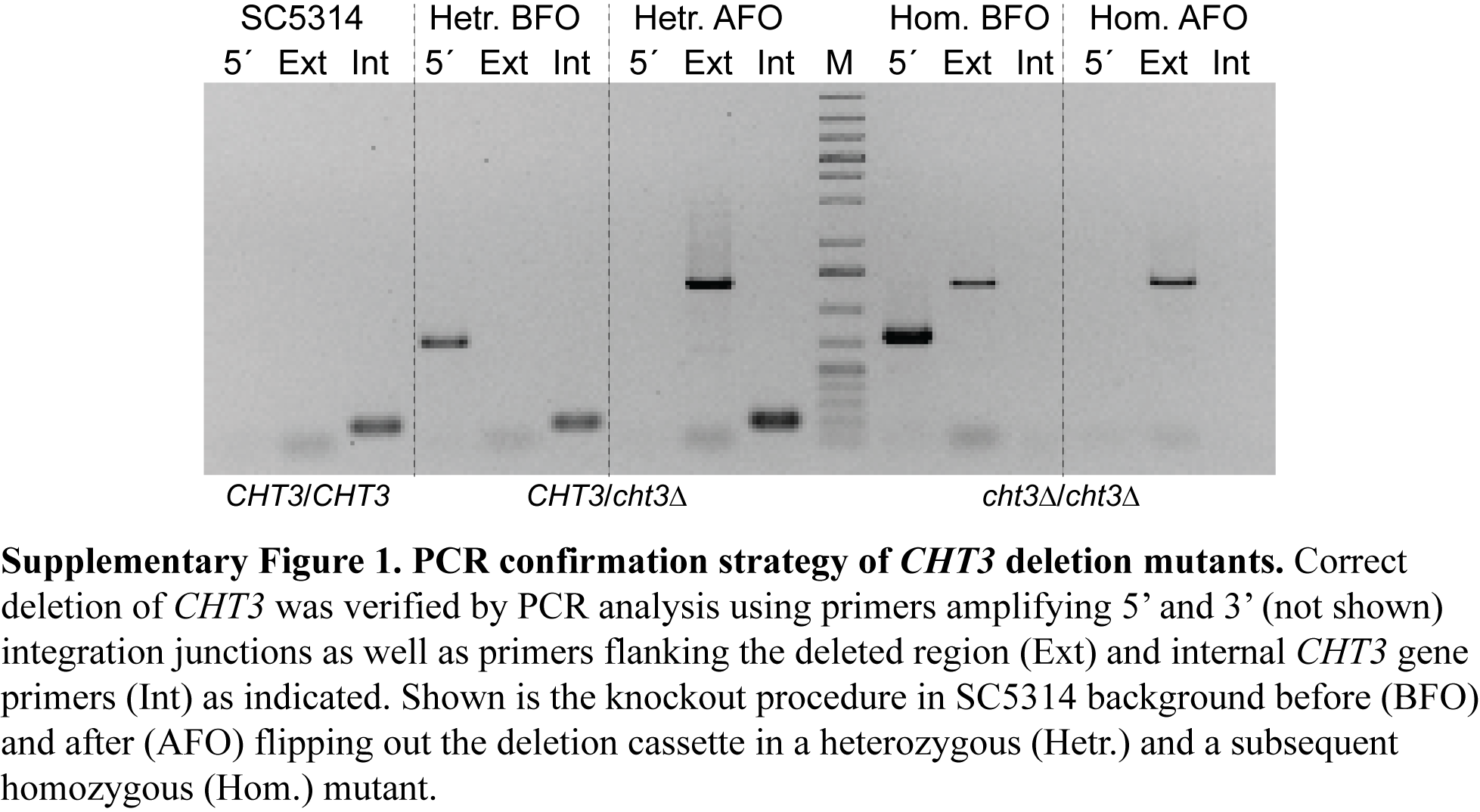

Supplement: Supplementary file 1 [file Image1.tif]

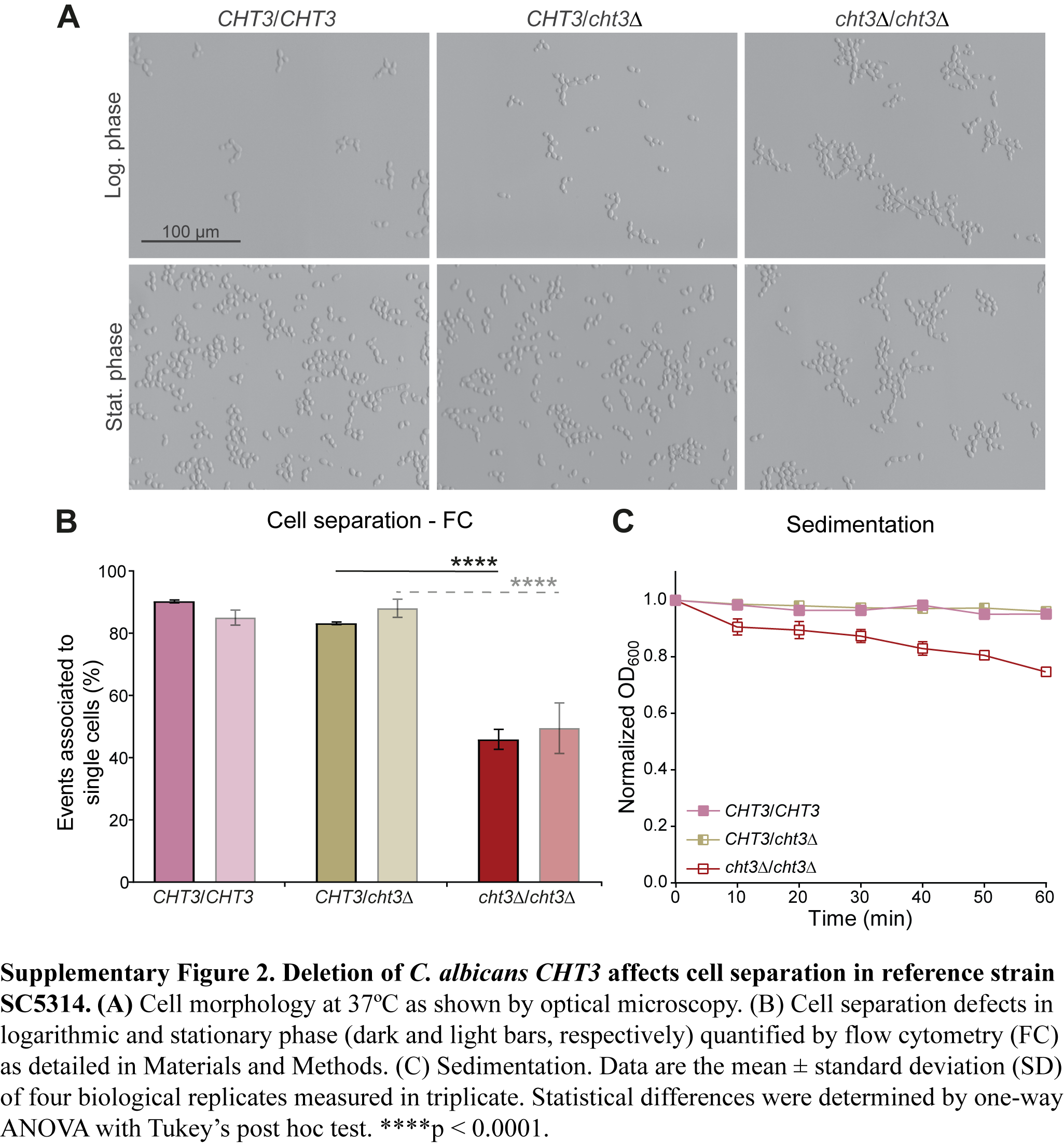

Supplement: Supplementary file 2 [file Image2.tif]

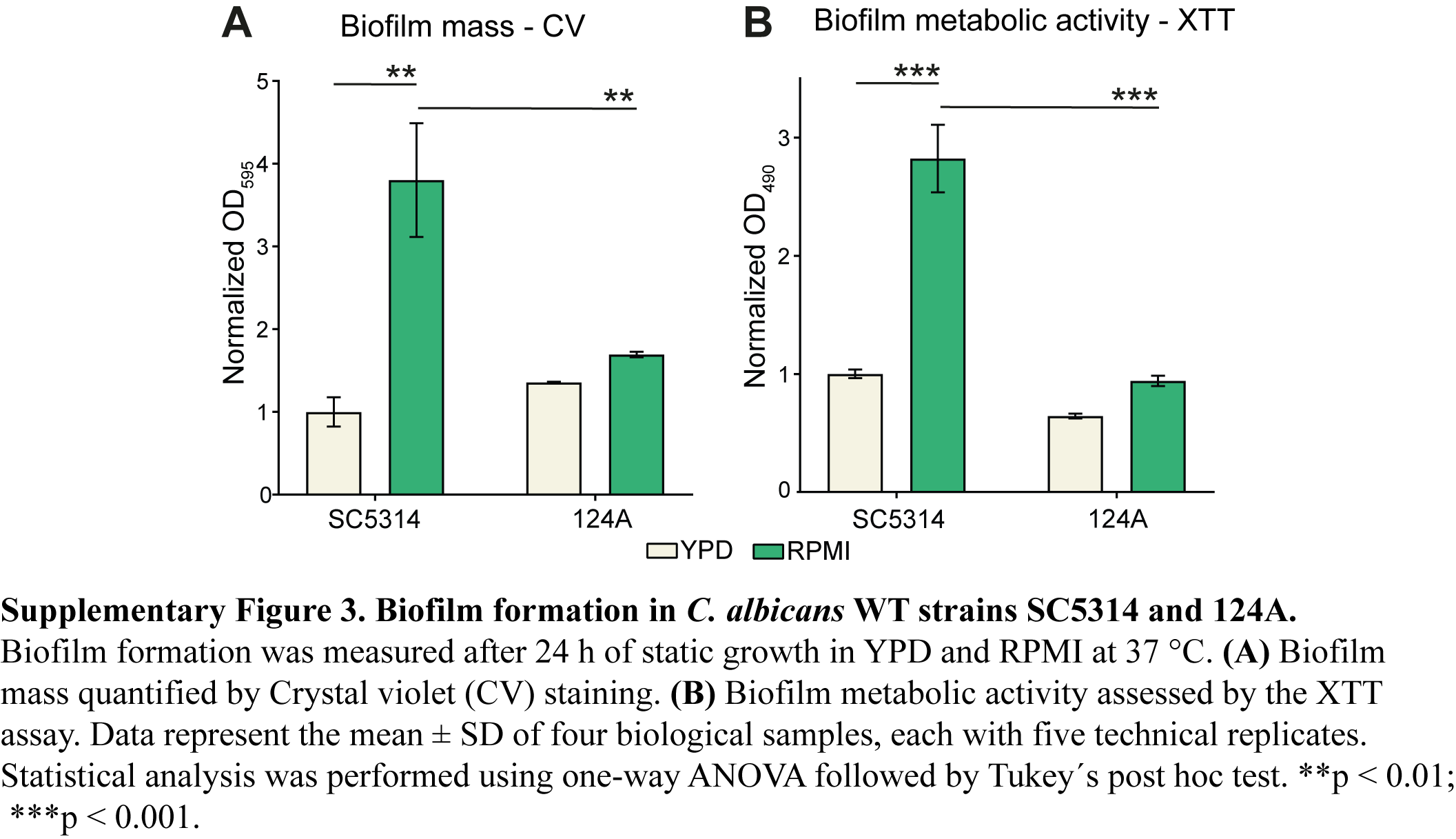

Supplement: Supplementary file 3 [file Image3.tif]

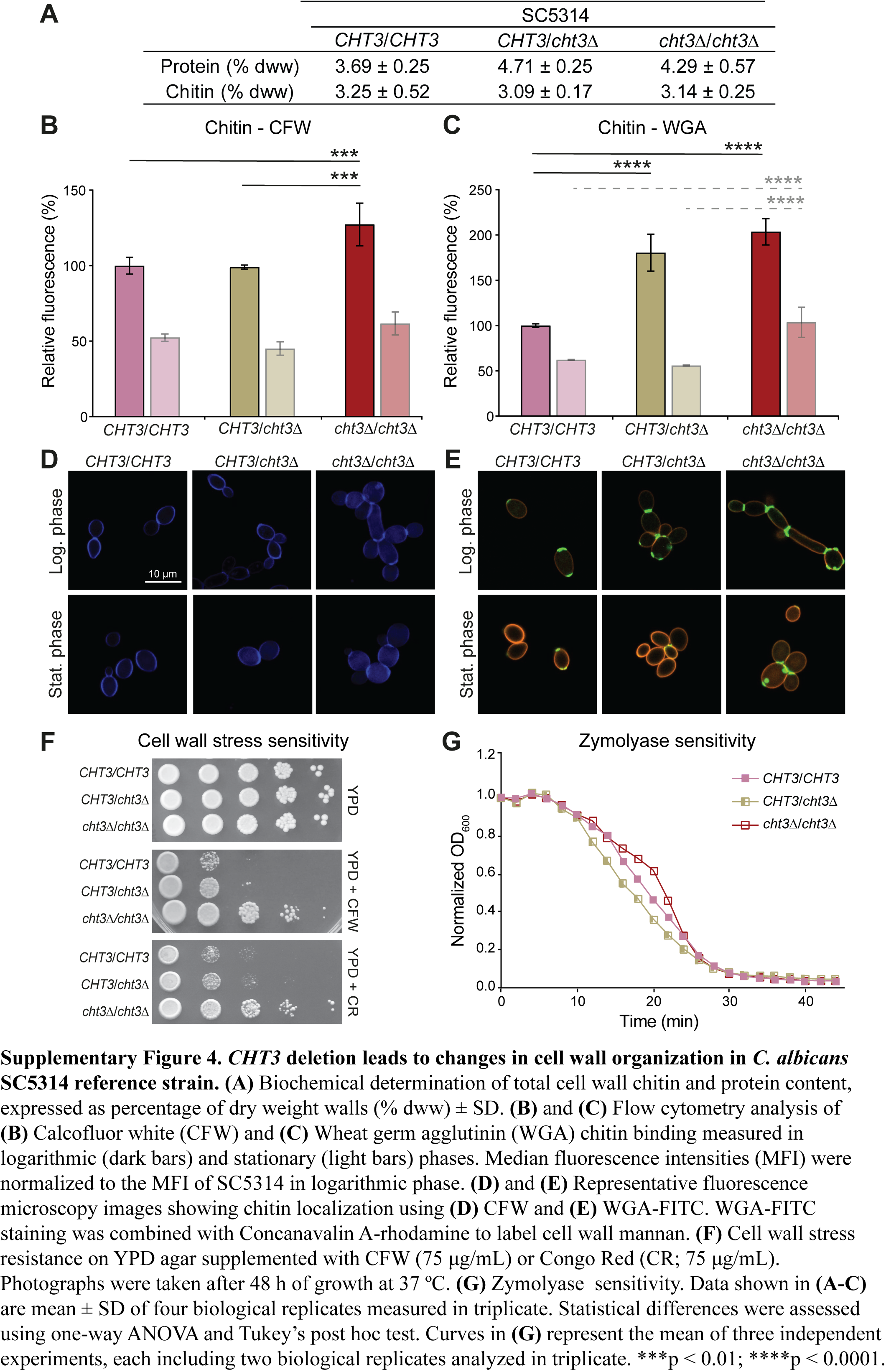

Supplement: Supplementary file 4 [file Image4.tif]

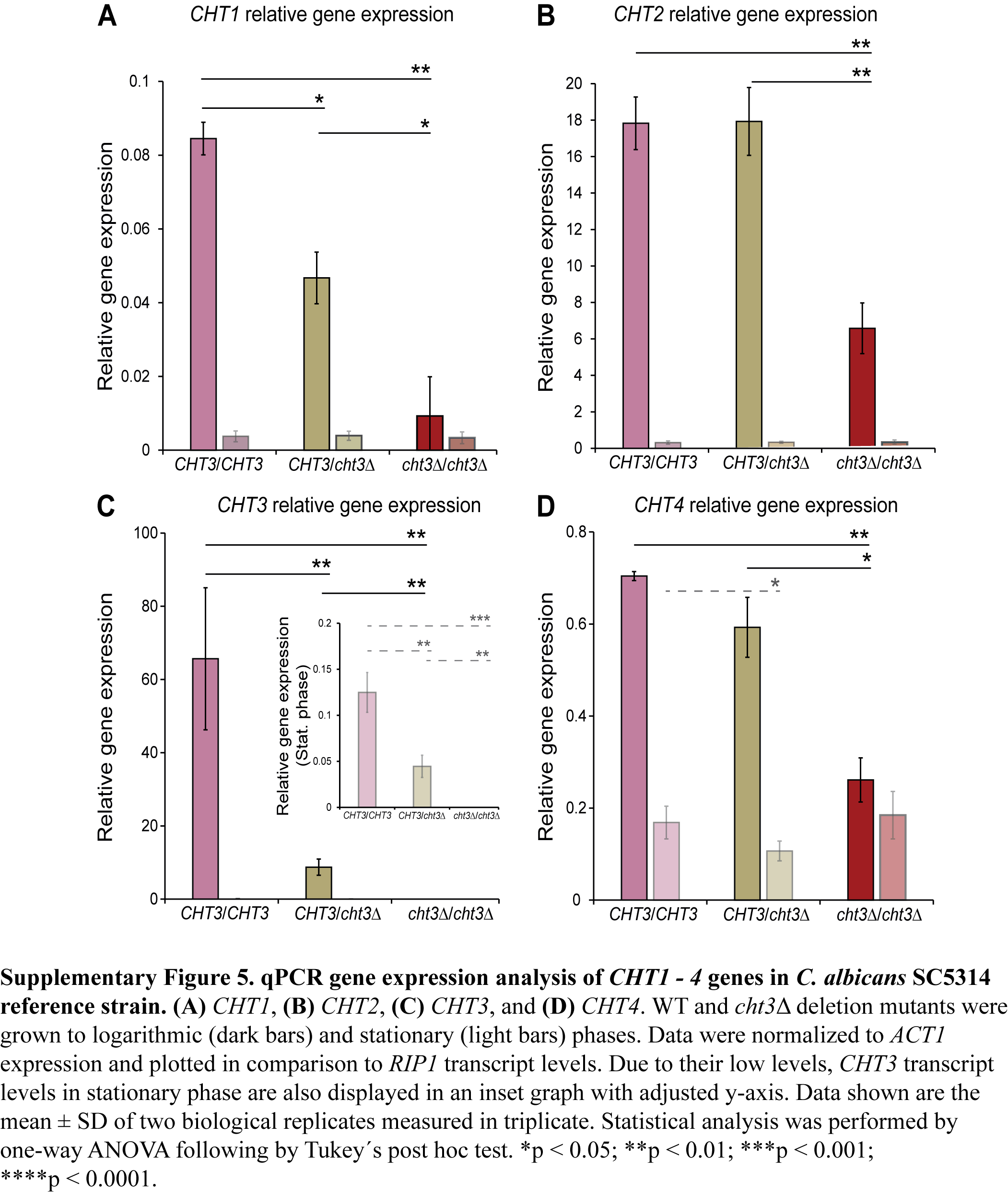

Supplement: Supplementary file 5 [file Image5.tif]

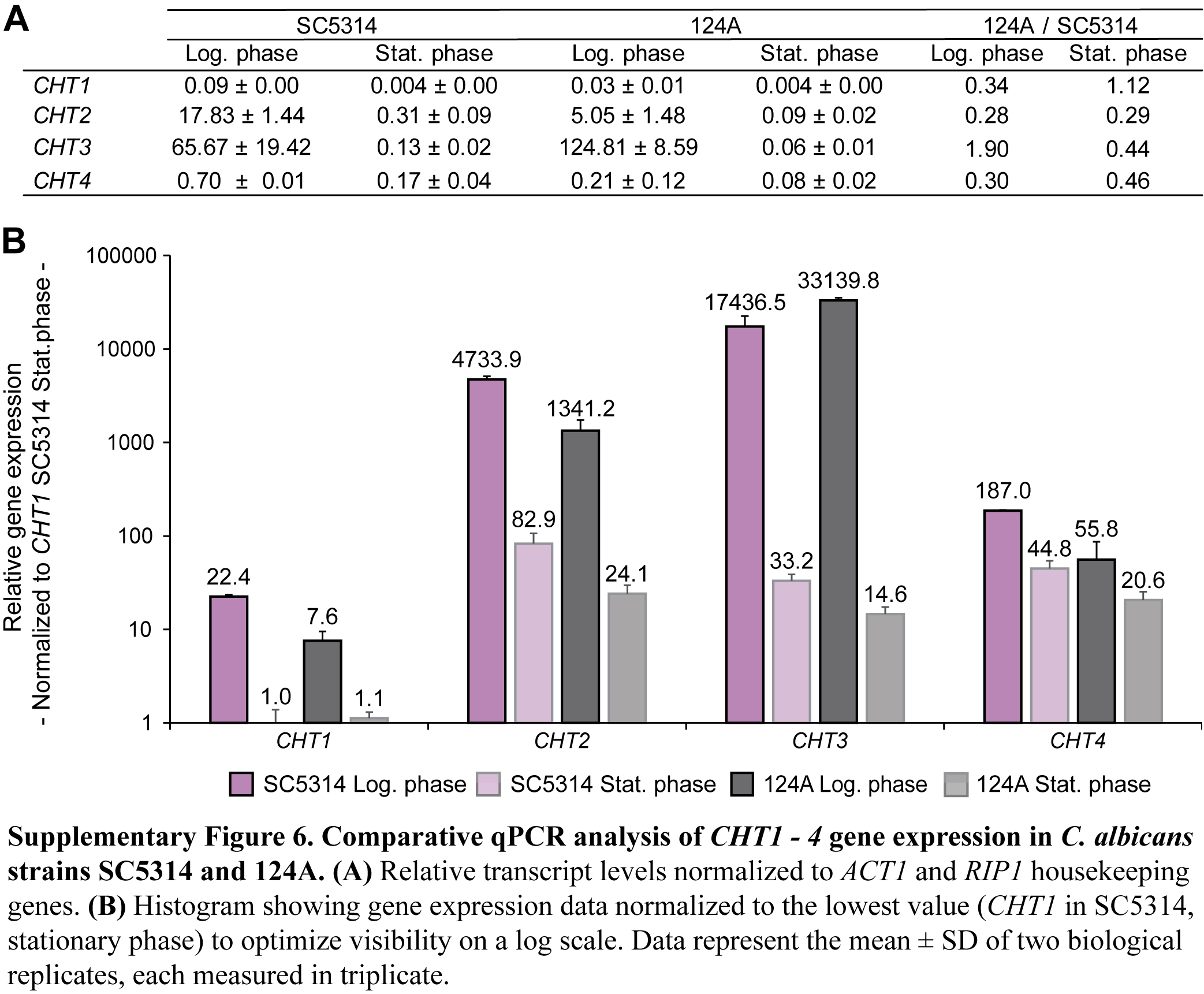

Supplement: Supplementary file 6 [file Image6.tif]

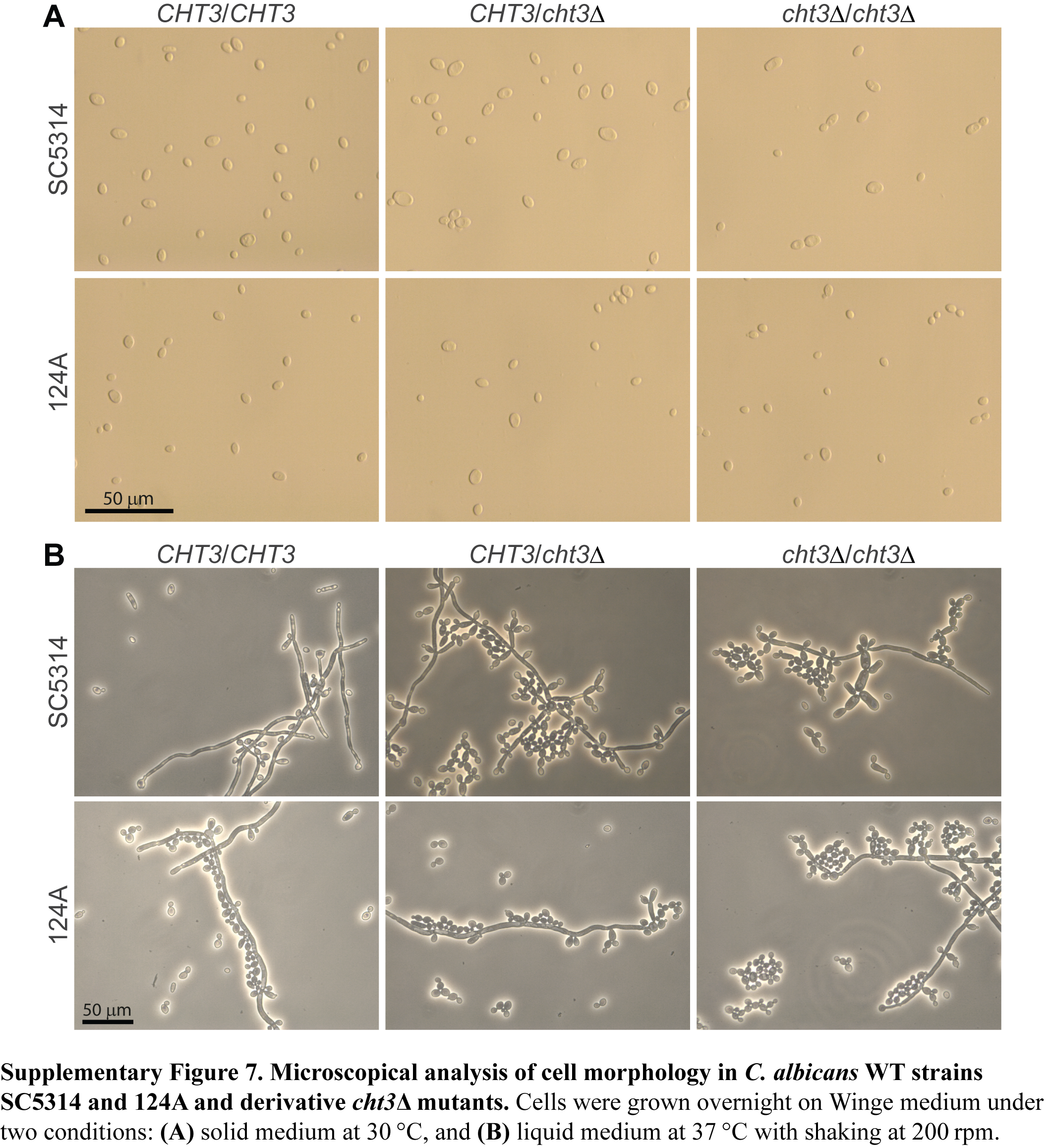

Supplement: Supplementary file 7 [file Image7.tif]
