# Supplementary material for: Loss of CHT3 in Candida albicans wild-type strains increases surface-exposed chitin and affects host-pathogen interaction
Source: Front Cell Infect Microbiol. 2025 Sep 5;15:1654710. doi: 10.3389/fcimb.2025.1654710 (PMC12446338; doi:10.3389/fcimb.2025.1654710)
Supplement: Supplementary file 8 [file Table1.docx]

**Supplementary data**

**Supplementary Table S1.** Strains used in this study.

| **Strain** | ***CHT3* genotype** | **Parental strain** | **Source** |
| --- | --- | --- | --- |
| SC5314 | *CHT3*/*CHT3* |  | See (Odds et al., 2004) |
| Hetr. mutant | *CHT3*/*cht3*Δ | SC5314 | This work |
| Hom. mutant 1 | *cht3*Δ/*cht3*Δ | Hetr. mutant | This work |
| Hom. mutant 2 | *cht3*Δ/*cht3*Δ | SC5314 | This work |
| 124A | *CHT3*/*CHT3* |  | (Sampaio et al., 2010) |
| Hetr. mutant | *CHT3*/*cht3*Δ | 124A | This work |
| Hom. mutant 1 | *cht3*Δ/*cht3*Δ | Hetr. mutant | This work |
| Hom. mutant 2 | *cht3*Δ/*cht3*Δ | 124A | This work |

**Supplementary Table S2.** Primers and CRISPR guide used in this study.

| **Oligonucleotide name** | **Sequence (5´- 3´)** | **Purpose** |
| --- | --- | --- |
| CHT3-KpnI_F | CACAGGTACCAGCTTTCCATGATGTTACG | Amplification and cloning of upstream *CHT3* flank in pSFS2 |
| CHT3-XhoI_R | CACACTCGAGCAAGTATAGCATTGGTGGG |  |
| CHT3-NotI_F | CACAGCGGCCGCTATCCCCGGTTCTATTCTG | Amplification and cloning of downstream *CHT3* flank in pSFS2 |
| CHT3-SacI_R | CACAGAGCTCCTCCTTATCCATGTACTCC |  |
| CHT3-ext_F | CGAGAACTTGAAAGGCAC | Checking CHT3 deletion mutants |
| CHT3-ext_R | GATGCAAAAATAATTCGTG |  |
| CHT3-int_F | CTGCTTCTTCTAGAGCC |  |
| CHT3-int_R | CTCCTGGTGAAGCTTTC |  |
| CHT3_guide | UUAAACAGCAAGUAUUUUGGGUUUUAGAGCUAUGCU | CRISPR-Cas9 sgRNA guide |
| ACT1_F | TACTCTGTCTGGATTGGTGG | Gene expression analysis |
| ACT1_R | GTGGTGAACAATGGATGGAC |  |
| RIP1_F | TGTCACGGTTCCCATTATGATATTT |  |
| RIP1_R | TGGAATTTCCAAGTTCAATGGA |  |
| CHT1_F | ACAATACGCCACACCAGTC |  |
| CHT1_R | TCCATCAAACCAACCAAACC |  |
| CHT2_F | TGCTCCACAATGTCCATACCC |  |
| CHT2_R | GTCAGCAAATTTGGACCAGG |  |
| CHT3_F | ATCCAGCGAGACAACCCCAG |  |
| CHT3_R | GCACTGGTGGTAGAAGTGGC |  |
| CHT4_F | AAATGCCACAACCATCACCCAATC |  |
| CHT4_R | ACATGTCCCCCATCCACCTA |  |

^1^Restriction enzyme recognition sites are underlined.

**Supplementary Table S3.** Growth rates (h^-1^) of *C. albicans* WT parental and *cht3*Δ mutants in YPD and Winge media at 30 ºC and 37 ºC.

| **Strains** | **30 °C** | | **37 °C** | |
| --- | --- | --- | --- | --- |
|  | **YPD** | **Winge** | **YPD** | **Winge** |
| SC5314 | 0.38 | 0.33 | 0.45 | 0.36 |
| Hetr. mutant | 0.39 | 0.33 | 0.44 | 0.32 |
| Hom. mutant 1 | 0.38 | 0.39 | 0.50 | 0.36 |
| Hom. mutant 2 | 0.41 | 0.37 | 0.48 | 0.33 |
| 124A | 0.37 | 0.37 | 0.48 | 0.41 |
| Hetr. mutant | 0.34 | 0.36 | 0.48 | 0.46 |
| Hom. mutant 1 | 0.35 | 0.36 | 0.47 | 0.40 |
| Hom. mutant 2 | 0.37 | 0.37 | 0.47 | 0.38 |
